# Inbreeding alters intersexual fitness correlations in *Drosophila simulans*.

**DOI:** 10.1002/ece3.1153

**Published:** 2014-08-05

**Authors:** Eoin Duffy, Richa Joag, Jacek Radwan, Nina Wedell, David J Hosken

**Affiliations:** 1 Institute of Environmental Science, Jagiellonian UniversityGronostawa 7, Krakow, Poland; 2 Centre for Ecology & Conservation, University of ExeterTremough, Penryn, TR10 9FE, U.K

**Keywords:** *Drosophila simulans*, inbreeding, intralocus sexual conflict, ontogenetic conflict

## Abstract

Intralocus sexual conflict results from sexually antagonistic selection on traits shared by the sexes. This can displace males and females from their respective fitness optima, and negative intersexual correlations (*r*_mf_) for fitness are the unequivocal indicator of this evolutionary conflict. It has recently been suggested that intersexual fitness correlations can vary depending on the segregating genetic variation present in a population, and one way to alter genetic variation and test this idea is via inbreeding. Here, we test whether intersexual correlations for fitness vary with inbreeding in *Drosophila simulans* isolines reared under homogenous conditions. We measured male and female fitness at different times following the establishment of isofemale lines and found that the sign of the association between the two measures varied with time after initial inbreeding. Our results are consistent with suggestions that the type of genetic variation segregating within a population can determine the extent of intralocus sexual conflict and also support the idea that sexually antagonistic alleles segregate for longer in populations than alleles with sexually concordant effects.

## Introduction

Intralocus sexual conflict occurs when an allele has opposing fitness effects depending on whether it is expressed in males or females (Rice 1996; Arnqvist and Rowe 2005; Bonduriansky and Chenoweth 2009). Negative intersexual fitness correlations (*r*_mf_) are the unequivocal signature of this sexual conflict (Chippindale et al. 2001; Bonduriansky and Chenoweth 2009), which can displace one or both sexes from their respective fitness optimum (Rice 1996; Arnqvist and Rowe 2005; Bonduriansky and Chenoweth 2009). The current interest in intralocus sexual conflict stems from its ubiquity and potential importance in many evolutionary processes including speciation, sexual selection, and the maintenance of genetic variation (reviewed in Bonduriansky and Chenoweth 2009; Van Doorn 2009; but see Pennell and Morrow 2013).

The empirical evidence for intralocus sexual conflict in both laboratory and natural populations is growing (Chippindale et al. 2001; Brommer et al. 2007; Foerster et al. 2007; Long and Rice 2007; Harano et al. 2010; Lewis et al. 2011; Mills et al. 2011; Punzalan et al. 2014). However, some studies have not detected intralocus sexual conflict, but instead find strong positive intersexual genetic correlations for components of male and female fitness (Hosken et al. 2003; Zajitschek et al. 2007; Poissant et al. 2010; Berg and Maklakov 2012). Recently, Long et al. (2012) proposed that the discrepancies between studies could result from differences in the genetic variation segregating in the populations under investigation. They found that high fitness males sired high fitness daughters in populations that were poorly adapted to their experimental conditions and low fitness daughters in well-adapted populations.

The explanation offered for this difference was that in well-adapted populations, most deleterious mutations had been eliminated by selection, such that genetic polymorphisms were maintained by some form of balancing selection, like sexually antagonistic selection (Long et al. 2012). Under this scenario, sexually antagonistic alleles are major contributors to genetic variation in fitness. On the other hand, in the less well-adapted populations where maladaptive alleles are abundant (because they had come from other populations in other environments), segregating genetic variation for fitness was suggested to be dominated by alleles with the same fitness effects for males and females – they either positively or negatively impact fitness regardless of gender. Recent modeling also shows that mutations which have sexually antagonistic effects inflate genetic variance in populations compared to mutations with sexually concordant effects (Connallon and Clark 2012). This result even holds when the conditions for balanced polymorphism are not met – in such case, sexual antagonism changes mutation–selection balance and inflates heterozygosity (Connallon and Clark 2012). In sum, these results suggest that population history, via its effects on the nature of genetic variation segregating in a population, is key to our understanding of intralocus sexual conflict and its effects on sexual selection.

If allelic effects act in the same direction for fitness in both sexes, then sexual and natural selection can be congruent, as predicted by “good genes” theory (Andersson 1994; Rowe and Houle 1996; Kuijper et al. 2012). Under such conditions, females' daughters may benefit from high genetic quality sires (i.e., those not burdened with maladapted alleles) (Zahavi 1975; Andersson 1986; Iwasa and Pomiankowski 1994; Rowe and Houle 1996; Radwan 2008). Furthermore, sexual selection should help populations reach adaptive peaks (Lande and Kirkpatrick 1988; Brooks et al. 2005). If, however, most genetic variation segregating in a population is maintained by sexually antagonistic selection, such benefits will not arise (Radwan 2008; Hosken et al. 2009; Connallon and Clark 2012). However, the nature of these effects may well vary due to changes in the genetic variation segregating in populations, although this idea has been subjected to few explicit tests (Long et al. 2012; Punzalan et al. 2014).

In previous tests of intersexual fitness and segregating genetic variation, alleles that were assumed to be deleterious were introduced into populations occupying different environments (e.g., Long et al. 2012). An alternative approach for testing the hypothesis that deleterious alleles can alter genetic variation and hence the sign of *r*_mf_ is by increasing the frequency of homozygotes in a population. Increasing the frequency of homozygotes increases the expression of deleterious recessive alleles, altering genetic variances (Fernández et al. 1995; Falconer and MacKay 1996; Wade et al. 1996; Whitlock and Fowler 1999). This can easily be achieved through inbreeding, which also erodes any benefits of overdominance.

Thus, one way to investigate the effects of segregating genetic variation on intralocus conflict, and its knock-on implications for sexual selection, is to inbreed populations and to test for changes in the intersexual correlation for fitness as inbreeding proceeds, and more and more deleterious recessive alleles are expressed. As large effect mutations tend to be both highly recessive (Willis 1999; Fox et al. 2008) and deleterious for both sexes (Sharp and Agrawal 2008, 2013; Whitlock and Agrawal 2009; Mallet et al. 2011), positive correlations between the sexes are expected during the initial stages of inbreeding (cf. the Long et al. (2012) results). Such large effect mutations are expected to be purged after their initial exposure (Charlesworth and Willis 2009; Jarzebowska and Radwan 2010) but as inbreeding proceeds, alleles with opposing sex-specific effects on fitness are expected to remain segregating (Chippindale et al. 2001; Connallon and Clark 2012), and then, genetic correlations between the sexes should change from positive to uncorrelated or negative as the contribution to standing genetic variation is predominately determined by alleles with sexually antagonistic effects.

To test these predictions, we measured male and female fitness during inbreeding in *D. simulans*. We generated intersexual genetic correlations for fitness to examine the sign of *r*_mf_ at different stages of inbreeding. Any changes in the sign of this association would indicate altered genetic variation because of changes in levels of homozygosity and could potentially support the notion that sexually antagonistic alleles are harder to purge than unconditionally deleterious alleles.

## Materials and Methods

### Base population

Flies had been maintained in six large population cages (ca. 800–1000 flies per cage) with overlapping generations and gene flow between cages for 6 years prior to beginning the current investigation. Cage populations cured of *Wolbachia* infection (Hoffmann et al. 1986) were established for another study, and from them, we established a single population using offspring from 378 inseminated, *Wollbachia-*cured females. This cage population was maintained at a size of ca. 800 flies with overlapping generations and housed in the laboratory at an ambient temperature between 23°C and 25°C for 40 generations, at which time we established isofemale lines used to test intersexual fitness correlations.

### Isolines and maintenance

To establish isofemale lines (isolines), 40 inseminated females were collected from the population cage and transferred to individual vials with ample food and allowed to oviposit for 48 h before they were removed. After 9–11 days, offspring emerged and were used to initiate isolines. Each isoline was subsequently maintained with *n* = 25 males and *n* = 25 females per line allowing for full-sib matings to occur within a single vial during generation 1. Further increase in inbreeding resulted from limited population size and proceeded at the minimum rate of 1/2*N* = 1% per generation, but most likely faster, due to for example, uneven male mating success and not all females contributing to the next generation. Such design achieved substantial initial increase in genome to identity by descent (25% under full-sib mating), but later, slower inbreeding allowed for more efficient selection against such exposed deleterious recessives than sib mating (Wang et al. 1999). Lines were inbred this way for four generations before conducting the first fitness assays. All isolines were housed at 25°C and at a 12:12 h light/dark cycle in 200-mL vials with approximately 30 mL of food media (1.2 L deionized water, 72 g oatmeal, 102 g brown sugar, 24 g yeast and 12 g agar and adding 0.75 g benzoic acid, 1 g methyl 4 hydroxybenzoate and 7 mL propionic acid as antimold and bacterial agents). After brothers and sisters within each isoline were allowed to mate and oviposit for 48 h, they were discarded and virgin offspring were collected after a 10- to 11-day development period. Virgin males and females were housed separately in 50-mL vials with ample food media (*n* = 5 individuals per vial) until sexually mature, 3- to 4-day posteclosion (Ashburner et al. 2005), and the process began again. Of the initial 40 isolines derived from the base population, seven had gone extinct before the first block of fitness assays and the remainder survived the duration of our experiment, for a final sample size of 33 isolines.

### Measuring fitness

Fitness for each isoline was assessed for ten individuals/sex over three blocks (hereafter inbreeding stage) which equated to short (five generations), medium (nine generations), and long (13 generations) periods of inbreeding, giving a total of 30 males and 30 females measured/isoline. This means that when isolines were assayed at each time point, they should harbor differing segregating genetic variation as a result of ongoing inbreeding relative to that present initially. For logistical purposes, assays for male and female fitness were not performed concurrently, but this should not consistently affect relative fitness ranks.

#### Female fitness

Female reproductive success (the number of offspring produced surviving to adulthood) was used as a measure of female fitness. This was scored as the total number of eclosed offspring produced by a single female (focal female) ovipositing over 7 days where each female was housed with two virgin sexually mature males collected from the base population cage the isolines were originally derived from (hereafter tester males). To collect virgin tester males from the base population cage, ten 100 mL food vials were placed in the cage for 24 h. Virgin tester males were then collected during the collection period of the virgin focal females following the same protocol described above.

During fitness assays, flies were transferred to new 35-mL vials (containing 10 mL of food) after 48 h in vials 1 and 2, after which time they were housed in a third vial for 72 h. Transfers were performed without anesthesia using aspiration to avoid any confounding effects on female ovipositing behavior or fertility due to CO2 anesthesia (Champion de Crespigny and Wedell 2008). After the final 72 h, all flies were removed and progeny allowed to develop. This measure of fitness includes an individual female's fecundity and the larval and early adult survivorship of her resulting offspring (Rundle et al. 2006; Delcourt et al. 2009). All offspring that eclosed from each vial were counted 8 days after the first eclosions were recorded to ensure only the offspring of focal females were scored – rather than risk including grand offspring (cf. Taylor et al. 2008; Sharma et al. 2010).

#### Male fitness

The fitness of males was determined by placing one sexually mature, virgin isoline (focal) male with four sexually mature virgin competitor males that were randomly chosen from an *ebony* recessive mutant *Wolbachia* free stock population in a mating vial. The isoline and *ebony* males competed for fertilizations with a randomly chosen sexually mature virgin *ebony* female.

Each cohort of flies was housed in three different vials over seven days as above. After the final 72 h, all flies were removed and vials incubated for offspring development. Offspring from each vial were counted on the eighth day after the first eclosions were observed as per female assay (Taylor et al. 2008; Sharma et al. 2010). The gray-black cuticle pigmentation of *ebony* flies allowed for easy scoring (using light microscopy) of wild-type relative to *ebony* offspring. The fitness of an isoline male was scored as the ratio of wild-type: *ebony* offspring in each vial. As *ebony* is a recessive allele, progeny have a wild-type phenotype if fertilization is by focal males (Ashburner et al. 2005). Using male competitiveness as a measure of male fitness has been used in other studies (Chippindale et al. 2001; Delcourt et al. 2009; Mills et al. 2011) and includes several components of fitness including male mating success, the subsequent productivity of the female the male mates with, and the survival to emergence of the male and female offspring in direct competition with half-sibling offspring sired by the *ebony* competitors (Delcourt et al. 2009).

### Statistical analysis

Our main question was: Does *r*_mf_ change at different stages of inbreeding in the isolines? We also asked was there evidence for intersexual variation in fitness between isolines? Prior to analysis, data were first *Z*-transformed using the mean and standard deviation for each inbreeding stage to express male and female fitness on the same scale. Means for each line were calculated and used to calculate Pearson's correlation coefficients between male and female fitness (*r*_mf_) at each inbreeding stage. In order to test the significance of differences in *r*_mf_, between different stages of inbreeding, we followed (Zar 1999) and used the following formula:


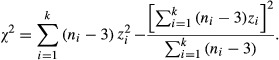


Each correlation coefficient (*r*_mf_) was transformed using Fisher's *Z* transformation (*z*_*i*_). Differences between correlation coefficients were tested using the preceding equation, which has a chi-square distribution with *k*–1 degrees of freedom. *n*_*i*_ represents the number of isolines assayed at each inbreeding stage (*n* = 33). To examine the total intersexual variation in fitness between different isolines, we fitted mixed-effect models using the lme4 package (Bates et al. 2011) in R version 3.0 (R Core Team 2013). Sex and inbreeding duration were included as fixed factors and isoline as a random factor, with isoline nested within stage of inbreeding. Interactions involving random terms were also coded as random effects. The significance of each term was tested against the full model with nonsignificant terms removed, and significant terms retained on the basis of likelihood ratio tests (compared against a *χ*^2^ distribution) to find a minimum adequate model (Crawley 2012) using standard maximum-likelihood estimation (Bolker et al. 2009). Our model indicated no evidence for overdispersion (ratio of residual deviance/residual df < 1.5 (0.86) (Ramsey and Schafer 2012). Outliers were identified and removed from female fitness data from the first (short) inbreeding stage (*n* = 2, *n* = 4 and *n* = 1 outliers from three separate isolines) and the third (long) inbreeding stage (*n* = 2 outliers from one isoline). Effect sizes (*η*^2^ and *η*_p_^2^) of isoline, sex, and their interaction were calculated from two-way mixed-effect ANOVAs performed for each stage of inbreeding using SPSS version 20 (IBM Corp. Released 2011; New York, U.S.A.).

## Results

The intersexual correlation coefficients for fitness (*r*_mf_) were found to be significantly different at the different stages of inbreeding (*χ^2^* = 6.972, *P* = 0.031), that is, as *P* is < 0.05, we can reject the H_0_: *ρ*_1_ = *ρ*_2_ = *ρ*_3_ where *ρ*_(1–3)_ is the population correlation coefficient at each inbreeding stage (short, medium, and long periods of inbreeding). We further investigated the difference between correlation coefficients by determining which correlation coefficients are different from which others, using a Tukey-type test as per the below equation, outlined below in Zar (1999):


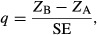


where *Z* is the *Z*_(B/A)_-transformed *r*_mf_ for each pair of correlation coefficients being compared, *q* is the studentized range, and SE is the standard error where 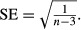 The correlation coefficient from lines after the short duration of inbreeding was significantly different from the coefficient obtained after the medium duration of inbreeding (*q =* 3.51, *P =* 0.014) and tended to be different from the coefficient obtained when inbreeding had been going on longest (*q* = 2.84, *P =* 0.075). Coefficients for the medium and longest duration of inbreeding were however not significantly different (*q* = 0.68, *P* = 0.496).

**Figure 1:**
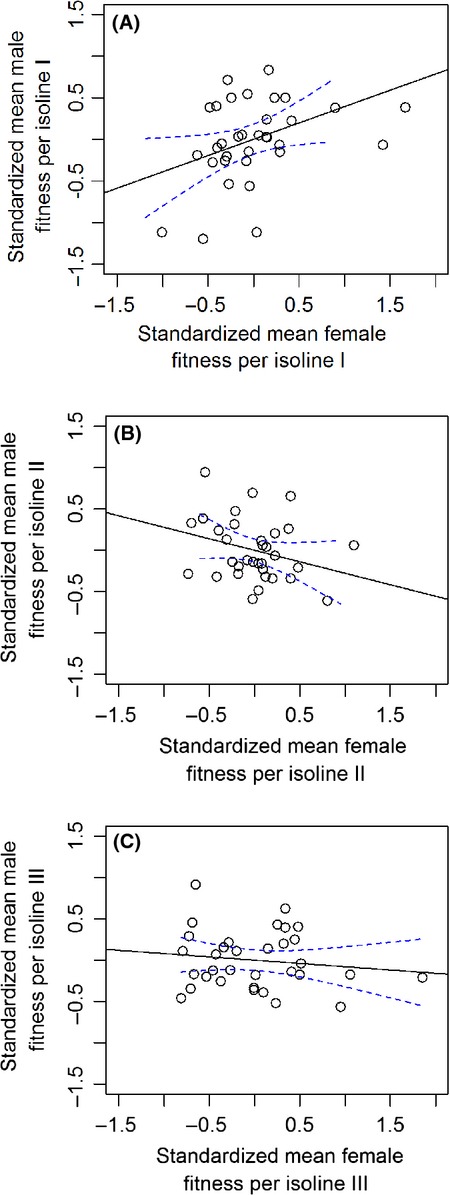
The intersexual fitness correlations at the three levels of inbreeding: (A) after 5 generations of inbreeding (short duration of inbreeding = I), (B) 9 generations of inbreeding (medium duration of inbreeding = II), and (C) 13 generations of inbreeding (long duration of inbreeding = III). For the short duration of inbreeding, (A) correlation of standardized line means revealed a positive male–female fitness association (df = 31, *P* = 0.039, *r* = 0.359. Linear regression *F*_1, 31_ = 4.6, *P* = 0.039, *r*^2^ = 0.101; *B =* 0.330, SE: 0.15). For the medium duration of inbreeding (intermediate inbreeding – B), correlation of standardized line means revealed a negative but nonsignificant male–female fitness association (df = 31, *P* = 0.145, *r* = −0.259. Linear regression *F*_1, 31_ = 2.2, *P* = 0.145, *r*^2^ = 0.037; *B =* −0.278, SE: 0.18). For the long duration of inbreeding (high inbreeding – C), correlation of standardized line means also revealed a negative but nonsignificant male–female fitness association (df = 31, *P* = 0.44, *r* = −0.140. Linear regression *F*_1,31_ = 0.62, *P* = 0.44, *r*^2^ = 0.012; *B =* −0.08, SE: 0.10). Blue lines around regression lines represent 95% confidence envelopes.

After the short period of inbreeding, *r*_mf_ was significantly positive (df = 31, *r*_mf_ = 0.3596, *P* = 0.039, Fig.1), and the association became negative, although not statistically significantly so, at later stages of inbreeding (medium inbreeding duration: df = 31, *r*_mf_ = −0.259, *P* = 0.145; long inbreeding duration: df = 31, *r*_mf_ = −0.140, *P* = 0.437, Fig.1). Although these latter associations are not significantly negative, they are still significantly lower than at the earlier stage, as we find correlation coefficients from the medium and long inbreeding stages tended to be significantly different from the short stage, our lowest level of inbreeding.

**Figure 2:**
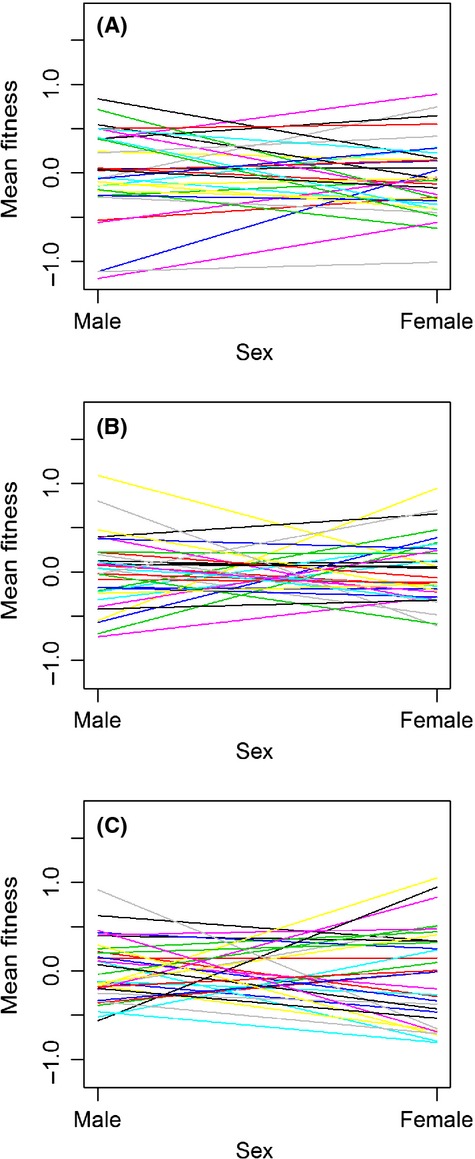
The sex (male–female) × isoline (genotype) interaction plots at the three levels of inbreeding: (A) after 5 generations of inbreeding (Short duration of inbreeding), (B) 9 generations of inbreeding (medium duration of inbreeding), and (C) 13 generations of inbreeding (long duration of inbreeding). Lines in plots represent standardized means of male (competitive success) and female (reproductive success) fitness for individual isolines (*n* = 33) where colors represent individual isolines.

In order to examine the sources of total intersexual variation in fitness, we fitted a three-way mixed-effect model, which indicated a highly significant sex-by-isoline by inbreeding stage interaction (likelihood ratio test, *χ*^2^_2_ = 23.173, *P* < 0.001). To get an insight into the nature of this three-way interaction, we ran two-way mixed-effect ANOVAs independently for each inbreeding duration. Terms were tested for significance as described above (sex: fixed effect, isoline: random effect). After the shortest duration of inbreeding, the sex-by-isoline interaction was nonsignificant (*F*_32, 594_ = 1.3383, *P* = 0.081), but there was a significant effect of isoline (*F*_32, 594_ = 2.39, *P* = 0.008). However, after the medium and long durations of inbreeding, the isoline-by-sex interactions were highly significant (medium duration: *F*_32*,* 594_ = 1.814, *P* = 0.005; long duration: *F*_32, 594_ = 2.601, *P* < 0.0001 Fig.2.), while the main effect of isoline at these stages of inbreeding was nonsignificant (medium stage: *F*_32*,* 594_ = 0.573, *P* = 0.940; long stage: *F*_32*,* 594_ = 0.791, *P* = 0.744). See Table S1 for effect sizes.

## Discussion

Intralocus sexual conflict has important implications for a range of phenomena, including speciation (Parker and Partridge 1998; Rice and Chippindale 2002), the evolution of sex chromosomes (Bull 1983; Rice 1987; Charlesworth 1991), the evolution of sex determination (Rice 1986; Kraak et al. 2002; Van Doorn and Kirkpatrick 2007), the regulation of gene expression (Ellegren and Parsch 2007; Mank et al. 2013), aging (Vieira et al. 2000; Bonduriansky et al. 2008), sexual selection (Pischedda and Chippindale 2006; Brommer et al. 2007), and sex allocation (Alonzo and Sinervo 2007; Katsuki et al. 2012). It is therefore crucial to understand why, despite selection for conflict resolution and multiple mechanisms of achieving this resolution (Hosken et al. 2009; Stewart et al. 2010; Pennell and Morrow 2013; but see Hosken 2011), intralocus sexual conflict is pervasive (Cox and Calsbeek 2009; Poissant et al. 2010).

While under certain circumstances sexually antagonistic selection can maintain genetic polymorphism (Rice and Chippindale 2001), recent modeling showed that a dynamic process of mutation–selection balance is more likely to maintain intersexual genetic correlations for sexually antagonistic traits (Connallon and Clark 2012). This latter mechanism arises because mutations that have an average deleterious effect but are sexually antagonistic segregate in populations for much longer than mutations deleterious for both sexes – because purifying selection against them is weaker. In principle, the same dynamics should result if the source of deleterious alleles is gene flow from populations adapted to different environments, rather than mutations (Long et al. 2012). If alleles underlying intralocus sexual conflict are maintained in populations transiently via mutation/migration–selection balance, the intersexual genetic correlation for fitness (*r*_mf_) should change with the rate of influx of maladapted alleles or with inbreeding, as it exposes recessive deleterious alleles. Indeed, we have found that the sign of the male–female fitness association changed with time from initial positive to negative (or zero depending on interpretation). This supposition is also supported by the three-way interaction between sex, isoline, and duration of inbreeding. As we argue below, these changes can be explained by selection against previously hidden genetic variation that has been exposed by inbreeding, and possibly, but less likely, due to subtle changes in the environments to which flies were subjected upon establishment of isolines (see below).

Inbreeding exposes recessive deleterious mutations, which are the most important source of inbreeding depression (Charlesworth and Willis 2009). Initial inbreeding, which can increase homozygosity by up to 25%, should reveal many deleterious recessives, and most of these are likely to have deleterious effects in both sexes (Mack et al. 2000; Sharp and Agrawal 2008, 2013; Whitlock and Agrawal 2009; Mallet et al. 2011). This should therefore generate a positive intersexual fitness correlation, as we observed. However, isolines are expected to show differences in the number and fitness effects of deleterious recessives fixed in them, due to initial differences in the genetic load of the founder females.

During further inbreeding of isolines, genome homozygosity should increase in smaller increments (Falconer and MacKay 1996). However, we expected selection to purge deleterious recessives from isolines as inbreeding proceeds, and those with sexually antagonistic effects should be eliminated at the slowest rate (Connallon and Clark 2012). Indeed, purging is most likely with slow inbreeding (Wang et al. 1999). Thus, we expected the change of the *r*_mf_ from positive to zero or even negative, which again is exactly what was observed. Many of the variants with opposing effects on male and female fitness should segregate or fix in isolines, so the negative genetic correlation between sexes should persist, as observed at the second and third test periods after relatively moderate (intermediate) and long durations of inbreeding. Furthermore, as deleterious recessives for both sexes are eliminated, inbreeding might initially lead to a decrease in genetic variance between lines. There is evidence for this as the isoline effect becomes nonsignificant at the intermediate level of inbreeding, while the line-by-sex interaction becomes highly significant there. This is also found after the longest duration of inbreeding, where the effect size of the interaction further increased (see Table S1). This is likely to have resulted from random assortment of male benefit-female harm alleles (and vice versa) between isolines. Thus, both the changes to *r*_mf,_ and the patterns of the sex-by-line interaction are consistent with changes in the load and sex specificity of deleterious recessives segregating in our flies. Deleterious (on average) recessives that have opposing effects on male and female fitness likely segregated for longer, as the average selection coefficient against them should be lower. This would increase the fitness variance between males and females due to the predominance of alleles with sexually discordant fitness effects.

The change in sign of *r*_mf_ associated with different levels of inbreeding could also be expected if some alleles become deleterious to both sexes due to the changes in the environment (Long et al. 2012). In our case, subtle changes in the rearing environment on establishment of isofemale lines could have such effects. As mentioned above (see Methods) prior to establishing isolines, the stock population cage was housed in a variable temperature regime (ca. 23–25°C) (and photoperiod) of the laboratory (i.e., not in an incubator), but for the duration of the experiment, isolines were housed in an incubator at 25°C (along with controlled humidity, 50% and photoperiod, 12:12 h light:dark). The slight temperature increase from the laboratory environment to a constant 25°C may have created an environment to which the isolines adapted, possibly revealing new genetic variation (Hoffman and Parsons 1991; Pigliucci et al. 1995; Kawecki et al. 1997; Hoffmann and Merilä 1999). In order to discriminate between the effect of inbreeding and temporal changes in *r*_mf_ due to, for example, subtle adaptations to laboratory conditions, *r*_mf_ should ideally be measured in outbred populations at each time point when isolines were measured. Unfortunately, our approach relying in estimating *r*_mf_ using isolines lines precluded that. However, we believe that due to prior adaptation of our stock population to laboratory conditions, these temporal effects would be relatively minor compared to the effect of inbreeding.

Overall, our results support recent findings suggesting that sexually antagonistic variation segregating in populations is dynamic in nature (Connallon and Clark 2012; Long et al. 2012) and can thus result in a change of *r*_mf_ depending on whether populations reached equilibrium with respect to mutation–selection or selection–migration balance. Processes such as environmental change or inbreeding, which move populations away from equilibrium, are thus likely to change the sign of *r*_mf_. The dynamic nature of this sexually antagonistic variation could potentially explain the range of findings reported across studies and species. Our results also caution against generalizations drawn from experiments using isofemale lines at various stages of inbreeding to interrogate the nature of sex-specific fitness components. Deleterious recessives, which may have limited impact on phenotypic variation in natural populations, may in fact act as a source of genetic variation in inbred populations, and as our result suggests, this may strongly affect the magnitude and sign of genetic correlation between sexes. However, such limitation does not concern hemiclonal lines (Chippindale et al. 2001; Pennell and Morrow 2013) or populations employing half-sib mating designs using populations with long histories of adaptation to defined experimental conditions (Delcourt et al. 2009).

## Supporting Information

**Table S1.** Effect sizes for sex, isoline and their interaction measured using partial Eta squared (*η*_p_^2^) and Eta squared (*η*^2^) for three levels of inbreeding (short, medium and long periods post establishment of isolines) where sex is a fixed effect and isoline is random (SS = sum of squares).
