# Supplementary material for: Inbreeding alters intersexual fitness correlations in Drosophila simulans
Source: Ecol Evol. 2014 Aug 5;4(17):3330–8. doi: 10.1002/ece3.1153 (PMC4228608; doi:10.1002/ece3.1153)
Supplement: Supplementary file 1 — Table S1. Effect sizes for sex, isoline and their interaction measured using partial Eta squared (ηp2) and Eta squared (η2) for three levels of inbreeding (short, medium and long periods post establishment of isolines) where sex is a fixed effect and isoline is random (SS = sum of squares). [file ece30004-3330-sd1.docx]

Table 1: Effect sizes for Sex, Isoline and their interaction measured using partial Eta squared (η_p_^2^) and Eta squared (η^2^) for three levels of inbreeding (short, medium and long periods post establishment of isolines) where sex is a fixed effect and Isoline is random (SS= sum of squares).

|  | | Short Inbreeding Stage | | | | | | Medium Inbreeding Stage | | | | | | Long Inbreeding Stage | | | | |
| --- | --- | --- | --- | --- | --- | --- | --- | --- | --- | --- | --- | --- | --- | --- | --- | --- | --- | --- |
| Source | | SS | | η^2^ | | η_p_^2^ | | SS | η^2^ | | | η_p_^2^ | | SS | | η^2^ | | η_p_^2^ |
| Sex | | 0.62 | | 0.001 | | 0.018 | | 9.33e-07 | 0 | | | 0 | | 0.62 | | 0.001 | | 0.01 |
|  | |  | |  | |  | |  |  | | |  | |  | |  | |  |
| Isoline | | 79.76 | | 0.142 | | 0.705 | | 31.96 | 0.051 | | | 0.364 | | 50.19 | | 0.087 | | 0.442 |
|  | |  | |  | |  | |  |  | | |  | |  | |  | |  |
| Sex x Isoline | | 33.37 | | 0.059 | | 0.069 | | 55.77 | 0.090 | | | 0.095 | | 64.33 | | 0.112 | | 0.123 |
| Error | | 561.60 | |  | |  | | 621.13 |  | | |  | | 574.21 | |  | |  |
|  |  | |  | |  | |  | | |  |  | |  | |  | |  | |
|  | |  | |  | |  | |  |  | | |  | |  | |  | |  |
|  | |  | |  | |  | |  |  | | |  | |  | |  | |  |
|  | |  | |  | |  | |  |  | | |  | |  | |  | |  |
|  | |  | |  | |  | |  |  | | |  | |  | |  | |  |
|  | |  | |  | |  | |  |  | | |  | |  | |  | |  |
|  | |  | |  | |  | |  |  | | |  | |  | |  | |  |
